# 固相萃取-高效液相色谱-串联质谱法同时测定燕窝中45种激素及其水平调查

**DOI:** 10.3724/SP.J.1123.2021.08008

**Published:** 2022-05-08

**Authors:** Dunming XU, Sanmei ZENG, Xuncai LIU, Luxiao WANG, Qunyan FAN, Xiaojiang ZHANG, Enhua FANG

**Affiliations:** 1.厦门海关技术中心, 福建 厦门 361026; 1. Technical Center, Xiamen Customs, Xiamen 361026, China; 2.厦门市燕之屋丝浓食品有限公司, 福建 厦门 361101; 2. Nest Technology Center, Yan Palace Seelong Food Co., Ltd., Xiamen 361101, China

**Keywords:** 高效液相色谱-串联质谱, 含量水平, 激素, 燕窝, high performance liquid chromatography-tandem mass spectrometry (HPLC-MS/MS), content level, hormones, edible bird's nest (EBN)

## Abstract

建立了固相萃取-高效液相色谱-串联质谱(SPE-HPLC-MS/MS)同时测定食用燕窝中皮质激素、雌激素、雄激素及孕激素等5类45种激素的多残留分析方法。采用乙腈-乙酸乙酯(1∶1, v/v)超声辅助提取、亲水亲脂平衡固相萃取柱净化,甲醇洗脱。分别在正、负电喷雾电离源、多反应监测模式下检测45种激素。正离子模式下的流动相为乙腈-0.1%甲酸水溶液,负离子模式下的流动相为乙腈-水,色谱柱为Phenomenex Kinetex C_18_柱(100 mm×2.1 mm, 2.6 μm)。在优化条件下,45种激素在0.20~20.0 μg/L范围内线性关系良好,相关系数(*R*^2^)≥0.9990,方法的检出限(LOD)为0.04~0.70 μg/kg,定量限(LOQ)为0.16~2.00 μg/kg。按三水平(2.0、4.0、20.0 μg/kg)进行加标回收试验,氟米龙、布地奈德、醛固酮、醋酸氟轻松、炔雌醇的回收率为40.2%~63.6%,可对这5种激素进行定性分析,其余40种激素的平均加标回收率为72.2%~112.3%,相对标准偏差(RSD)为2.5%~11.6%,该方法可对40种激素进行准确定性定量,精密度好,灵敏度高,简便、快速。从2017~2021年,通过研究建立的方法对来自马来西亚、印度尼西亚、泰国和越南等国家的1021个燕窝样品进行监测,仅勃地酮、雄烯二酮、孕酮有检出(大于检出限),其他激素均小于检出限。孕酮检出率为100%,勃地酮、雄烯二酮检出率分别为79%和89%, 3种激素含量范围分别为0.097~3.58、0~0.096和0~1.77 μg/kg。与同为动物源性食品的鸡蛋、纯牛奶、乳制品相比,所有测定的鸡蛋样品中均检出雄烯二酮,含量比其他3类产品略高;勃地酮在4类产品中的含量差别不大,均为微量;孕酮含量在鸡蛋中最高,其次是纯牛奶,燕窝中含量最低。研究结果表明,食用燕窝带入的激素种类少,含量低,对健康影响小。

## Abstract (en)

Edible bird's nests (EBNs), a food of animal origin, are temporary nests built by swiftlets to foster their offspring. As EBNs and their products are widely accepted by consumers, the safety of hormones in EBNs has also received increasing attention. The establishment of a method for hormone analysis in EBNs and the investigation of hormone levels based on the analytical method are the most effective measures to eliminate any safety concerns. In this study, a multi-residue method was developed for the simultaneous determination of 45 hormones in EBNs, including estrogens, progesterones, androgens, and cortical hormones. EBN samples (1.0 g) were weighed into 50 mL polypropylene centrifuge tubes and mixed with 8 mL of pure water. Then, the samples were extracted twice with 15 mL of acetonitrile and ethyl acetate (1∶1, v/v) under ultrasonic-assisted conditions for 30 min, and the protein in the EBN samples was precipitated at 4000 r/min for 5 min. The clear supernatants were loaded onto a hydrophilic-lipophilic balanced (HLB) SPE column, which was previously activated with methanol (3 mL) followed by pure water (3 mL). The cartridge was washed with 3 mL of pure water and 3 mL of 50% methanol aqueous solution. The hormones were eluted with 3 mL of methanol. A rapid analysis was performed using high performance liquid chromatography-tandem mass spectrometry (HPLC-MS/MS). The hormones in the extracting solution were separated on a Phenomenex Kinetex C_18_ column (100 mm×2.1 mm, 2.6 μm) and eluted by gradient elution with acetonitrile-0.1% formic acid aqueous solution (ESI^+^) or acetonitrile-water (ESI^-^). Qualitative and quantitative analyses were performed using the multiple reaction monitoring (MRM) mode. The HPLC-MS/MS results showed good linearity in the range of 0.20-20.0 μg/L with correlation coefficients (*R*^2^) ≥0.9990. For the 45 hormones, the limits of detection (LODs, *S/N*≥3) were 0.04-0.70 μg/kg and the limits of quantification (LOQs, *S/N*≥10) were 0.16-2.00 μg/kg. The recoveries of five hormones, namely, fluorometholone, budesonide, aldosterone, fluocinonide, and ethinylestradiol, were 40.2%-63.6%. Owing to the low recoveries, this method might be suitable only for qualitative testing of the five hormones. The recoveries of the other 40 target analytes were between 72.2% and 112.3% at spiked levels of 2.0, 4.0, and 20.0 μg/kg with relative standard deviations (RSDs) of 2.5%-11.6%. The method is characterized by easy operation, rapidness, high precision, and high sensitivity for the 40 compounds. Thus, this method is suitable for determination of the 40 hormones from EBNs for qualitative testing and quantitation. The proposed method was used to analyze 1021 EBN samples from Malaysia, Indonesia, Thailand, and Vietnam from 2017 to 2021. Only three hormones, progesterone, boldenone, and androstenedione, were identified in the EBN samples, while the levels of the other hormones were lower than their individual LODs. The detected rates of progesterone, boldenone, and androstenedione were 100%, 79%, and 89%, respectively. The contents of progesterone, boldenone, and androstenedione in the EBN samples were 0.097-3.58 μg/kg, 0-0.096 μg/kg and 0-1.77 μg/kg, respectively. The levels of hormones in EBNs were compared with those in eggs, pure milk, and dairy products, which were also animal-derived foods. Androstenedione was detected in all egg samples monitored, and its content was higher than that in EBN samples, pure milk, and dairy products. The content of boldenone was similar among the four products investigated in this study. Based on risk assessment using progesterone, the dietary intake was found to be 3.54 μg/d from milk >1.09 μg/d from eggs >0.0030 μg/d from EBNs. The results showed that the levels of hormones in EBNs were much lower than those in eggs, milk, and dairy products for daily consumption. Based on this investigation, the health effect of the hormones in EBNs may be insignificant.

燕窝,又名燕菜、燕蔬菜及燕根,是由部分雨燕科金丝燕用其唾液及绒羽等混合凝结筑成用于繁衍后代的巢窝,含有丰富的氨基酸、蛋白质、唾液酸及微量的矿物质元素等^[1,2]^,具有抗氧化^[3⇓-5]^、改善免疫^[6]^、延缓衰老^[7]^、抗病毒^[8,9]^、营养神经^[10]^、防止胰岛素抵抗^[11]^等功效,在《本草纲目拾遗》中有“大养肺阴,化痰止咳,补而能清,为调理虚损老擦之圣药”的记载。燕窝自引入中国以来就被奉为高档保健食品,广受人们青睐。燕窝作为一种动物源性食品,其中激素的安全性问题也受到更多人的关注。通过索引,Ma等^[12]^采用免疫技术对燕窝中6种激素进行测定,国内外有关采用液相色谱-串联质谱技术(LC-MS/MS)测定燕窝中激素含量的文献报道甚少,鉴于此,建立一种准确可靠的燕窝中多种激素同时确证的分析方法非常必要。

研究表明,长期摄入激素对于人体健康存在一定风险与隐患。如雌激素其化学稳定性高,不易降解,通过污水处理厂或养殖场废弃物进入环境后,在水体、土壤系统中将发生吸附、迁移等行为^[13]^,可在动物机体内蓄积,并通过食物链传递进入人体,干扰内分泌系统,并引起发育、生殖、行为等方面的异常变化^[14⇓-16]^。欧盟委员会禁止使用雌激素作为促生长剂用于动物育肥^[17]^,我国农业部公告250号^[18]^也规定食品动物中禁止使用己二烯雌酚、己烯雌酚、己烷雌酚及其盐、酯。目前,在环境领域研究较多的是雌激素,而对其他几种类固醇激素的研究偏少,且多是集中在少数几种物质上的基础研究,对于动物源性食品中激素的研究主要针对肉制品、牛奶、蛋等食品。我国现行的动物源性食品中激素的检测标准近30个,常用检测痕量残留激素的分析方法有主要有气相色谱-质谱法(GC-MS)^[19,20]^、电化学法^[21,22]^、酶免疫分析法^[23]^、放射免疫法^[24]^、高效液相色谱法^[25]^、液相色谱-三重四极杆质谱法^[26⇓⇓⇓-30]^等。其中,酶免疫分析法可测定范围较窄且易存在假阳性结果和交叉污染情况,适用于检测常见、单一性激素;高效液相色谱法对热稳定差、高沸点物质有较好分离度,但是紫外检测器灵敏度及准确性存在一定限制;GC-MS可对多种激素同时定性定量分析,但激素具有不易挥发及热不稳定性等特点,多数需要衍生,目前尚未有一种衍生试剂可对所有激素进行衍生化,而且衍生化过程还会出现副反应,使分析底物复杂化,过程繁琐耗时。LC-MS/MS因具有检测灵敏度高、选择性和特异性好、分析时间短等特点,是目前应用最广泛的激素残留定量分析方法之一^[31⇓⇓⇓-35]^。

本研究以燕窝为对象,以LC-MS/MS技术为检测手段,建立了燕窝中皮质激素、雌激素、雄激素及孕激素类等45种激素的测定方法,该方法操作简单,灵敏度高,准确度好,对后续燕窝食品安全的监管,以及回应社会对燕窝中激素安全性的担忧具有重要参考意义。

## 1 实验部分

### 1.1 仪器与试剂

Agilent 1290超高效液相色谱仪(美国Agilent公司), API5500三重四极杆质谱仪(美国AB公司), CPX5800H超声仪(美国Branson Ultrasonics公司), Supelco 24位固相萃取仪(美国Merck公司), N-EVAP-24氮吹仪(美国Organomation公司), MS3 Basic涡旋振荡器(德国IKA公司), TDL-50B离心机(中国上海安亭科学仪器厂), Milli-Q Reference超纯水器(美国Millipore公司), CPA225D电子天平(德国Sartorius公司)。

群勃龙等45种标准品(纯度≥98%,德国Dr. Ehrenstorfer公司、美国药典公司、英国药典公司、加拿大TRC公司)分别以甲醇溶解配制成质量浓度为100 mg/L的标准储备液,储存于-20 ℃冰箱中,使用时根据需要配成不同质量浓度的中间混合标准溶液及标准工作液。甲醇、乙腈、乙酸乙酯均为色谱纯(美国Merck公司),其他试剂为分析纯(国药集团化学试剂有限公司); 3 mL/60 mg亲水-亲脂平衡固相萃取柱(HLB,美国Waters公司)。本研究共收集1021份燕窝样品,来自马来西亚、印度尼西亚、泰国和越南等国家,测定时间2017~2021年。

### 1.2 色谱条件

#### 1.2.1 正离子模式

色谱柱:Phenomenex Kinetex C_18_色谱柱(100 mm×2.1 mm, 2.6 μm);柱温:35 ℃;流动相:A相为0.1%甲酸水溶液,B相为乙腈;流速:0.3 mL/min。梯度洗脱程序:0~2.0 min, 70%A; 2.0~2.1 min, 70%A~50%A; 2.1~7.0 min, 50%A~36%A; 7.0~7.1 min, 36%A~16%A; 7.1~8.6 min, 16%A~0%A; 8.6~10.6 min, 0%A; 10.6~11.0 min, 0%A~70%A; 11.0~14.0 min, 70%A。进样量:10 μL。

#### 1.2.2 负离子模式

色谱柱:Phenomenex Kinetex C_18_色谱柱(100 mm×2.1 mm, 2.6 μm);柱温:35 ℃;流动相:A相为水,B相为乙腈;流速:0.3 mL/min;梯度洗脱程序:0~1.5 min, 98%A~65%A; 1.5~3.5 min, 65%A; 3.5~3.8 min, 65%A~35%A; 3.8~5.0 min, 35%A; 5.0~5.3 min, 35%A~5%A; 5.3~7.0 min, 5%A; 7.0~7.1 min, 5%A~98%A; 7.1~10.0 min, 98%A。进样量:10 μL。

### 1.3 质谱条件

电喷雾电离源,正或负离子模式(ESI^+^或ESI^-^);离子源温度:650 ℃;雾化气压力:413.7 kPa;干燥气压力:413.7 kPa;气帘气压力:275.8 kPa;每个离子对驻留时间:10 ms。每种化合物的保留时间、检测离子对、去簇电压(DP)、碰撞能量(CE)等质谱参数见表1。

**表1:** 45种激素的质谱参数

No.		Compound	CAS No.		*t*_R_/min		Parent ion (*m/z*)		Product ion (*m/z*)		DP/V		CE/eV		Ionization mode
1		triamcinolone (曲安西龙)	124-94-7		2.52		439.2		363.3^*^		-66		-20		[M-H]^-^
									393.2		-66		-14		[M-H]^-^
2		aldosterone (醛固酮)	52-39-1		3.05		405.2		331.1^*^		-60		-30		[M-H]^-^
									359.1		-60		-17		[M-H]^-^
3		betamethasone (倍他米松)	378-44-9		3.20		393.2		337.3^*^		60		18		[M+H]^+^
									355.2		60		18		[M+H]^+^
4		fluoxymesterone (氟甲睾酮)	76-43-7		3.33		337.2		131.0^*^		40		47		[M+H]^+^
									241.3		40		33		[M+H]^+^
5		prednisolone (泼尼松龙)	50-24-8		3.42		405.2		329.1^*^		-40		-24		[M-H]^-^
									359.0		-40		-17		[M-H]^-^
6		estriol (雌三醇)	50-27-1		3.43		287.1		170.9^*^		-100		-50		[M-H]^-^
									144.9		-100		-57		[M-H]^-^
7		prednisone (泼尼松)	53-03-2		3.46		403.2		327.1^*^		50		-19		[M-H]^-^
									357.1		50		-13		[M-H]^-^
8		hydrocortisone (氢化可的松)	50-23-7		3.50		407.2		331.2^*^		-26		-24		[M-H]^-^
									361.1		-26		-17		[M-H]^-^
9		boldenone (勃地酮)	846-48-0		3.62		287.2		121.0^*^		45		31		[M+H]^+^
									135.0		45		21		[M+H]^+^
10		cortisone (可的松)	53-06-5		3.62		405.2		329.0^*^		-30		-23		[M-H]^-^
									359.2		-30		-13		[M-H]^-^
11		19-nortestosterone (诺龙)	434-22-0		3.73		275.2		109.2^*^		50		33		[M+H]^+^
									134.9		50		28		[M+H]^+^
									257.3		50		24		[M+H]^+^
12		norethindrone (炔诺酮)	68-22-4		4.03		299.2		109.1^*^		20		32		[M+H]^+^
									231.1		20		24		[M+H]^+^
13		21-hydroxyprogesterone (21-羟基孕酮)	64-85-7		4.09		331.2		97.0^*^		140		28		[M+H]^+^
									109.0		140		34		[M+H]^+^
14		6*α*-methylprednisolone (甲基泼尼松龙)	83-43-2		4.19		419.2		343.1^*^		-16		-26		[M-H]^-^
									373.3		-16		-17		[M-H]^-^
15		androstenedione (雄烯二酮)	63-05-8		4.30		287.2		97.1^*^		110		31		[M+H]^+^
									109.1		110		31		[M+H]^+^
16		methyltestosterone (甲睾酮)	58-18-4		4.33		303.2		97.1^*^		50		32		[M+H]^+^
									109.1		50		35		[M+H]^+^
17		17*α*-hydroxyprogesterone (17*α*-羟基孕酮)	68-96-2		4.35		331.2		97.2^*^		100		33		[M+H]^+^
									109.2		100		38		[M+H]^+^
18		dexamethasone (地塞米松)	50-02-2		4.40		437.2		361.1^*^		-25		-25		[M-H]^-^
									391.1		-25		-16		[M-H]^-^
19		flumethasone (氟米松)	2135-17-3		4.46		455.2		379.1^*^		-40		-27		[M-H]^-^
									409.2		-40		-17		[M-H]^-^
20		beclomethasone (倍氯米松)	4419-39-0		4.67		453.2		377.2^*^		-40		-21		[M-H]^-^
									407.1		-40		-18		[M-H]^-^
No.	Compound			CAS No.		*t*_R_/min		Parent ion (*m/z*)		Product ion (*m/z*)		DP/V		CE/eV	Ionization mode
21	levonorgestrel (左炔诺孕酮)			797-63-7		4.70		313.2		108.9^*^		41		39	[M+H]^+^
										245.3		20		24	[M+H]^+^
22	5*α*-androstan-17*β*-ol-3-one (雄烯醇酮)			5295-66-9		4.74		291.1		159.2^*^		166		36	[M+H]^+^
										255.4		166		24	[M+H]^+^
23	triamcinolone acetonide (醋酸曲安奈德)			3870-07-3		4.87		479.2		337.1^*^		-30		-34	[M-H]^-^
										413.1		-30		-31	[M-H]^-^
24	medroxyprogesterone (甲羟孕酮)			520-85-4		4.98		345.3		97.0^*^		40		33	[M+H]^+^
										123.2		40		36	[M+H]^+^
25	stanozolol (康力龙)			10418-03-8		5.06		329.3		81.1^*^		58		84	[M+H]^+^
										95.0		58		52	[M+H]^+^
26	mesterolone (甲氢睾酮)			1424-00-6		5.11		305.2		173.3^*^		180		30	[M+H]^+^
										269.3		180		24	[M+H]^+^
										229.2		180		26	[M+H]^+^
27	17*β*-estradiol (17*β*-雌二醇)			50-28-2		5.12		271.1		183.2^*^		-160		-53	[M-H]^-^
										144.8		-160		-52	[M-H]^-^
28	fluocinolone acetonide (醋酸氟轻松)			67-73-2		5.13		497.2		355.2^*^		-33		-35	[M-H]^-^
										431.2		-33		-28	[M-H]^-^
29	estrone (雌酮)			53-16-7		5.28		269.1		144.9^*^		-30		-18	[M-H]^-^
										158.9		-30		-26	[M-H]^-^
30	fluorometholone (氟米龙)			426-13-1		5.34		421.2		254.8^*^		-22		-29	[M-H]^-^
										354.9		-22		-22	[M-H]^-^
31	fludrocortisone acetate (醋酸氟氢可的松)			514-36-3		5.41		467.3		349.0^*^		-30		-36	[M-H]^-^
										421.1		-30		-19	[M-H]^-^
32	17*α*-estradiol (17*α*-雌二醇)			57-91-0		5.43		271.2		144.9^*^		-160		-50	[M-H]^-^
										182.9		-160		-55	[M-H]^-^
										239.1		-160		-54	[M-H]^-^
33	trenbolone acetate (醋酸群勃龙)			10161-34-9		5.62		313.2		253.3^*^		50		29	[M+H]^+^
										271.0		50		28	[M+H]^+^
34	megestrol-17-acetate (醋酸甲地孕酮)			595-33-5		5.90		385.2		267.1^*^		90		25	[M+H]^+^
										325.2		90		19	[M+H]^+^
35	progesterone (孕酮)			57-83-0		5.91		315.2		97.0^*^		35		31	[M+H]^+^
										109.0		35		32	[M+H]^+^
										297.3		35		22	[M+H]^+^
36	danazol (达那唑)			17230-88-5		5.95		338.2		120.2^*^		12		41	[M+H]^+^
										148.2		8		34	[M+H]^+^
37	chloromadinone 17-acetate (乙酸氯地孕酮)			302-22-7		6.06		405.2		309.1^*^		90		23	[M+H]^+^
										345.2		90		19	[M+H]^+^
38	melengestrol acetate (甲烯雌醇乙酸脂)			2919-66-6		6.16		397.2		279.2^*^		11		27	[M+H]^+^
										337.1		8		20	[M+H]^+^
39	medroxyprogesterone-17-acetate (醋酸甲羟孕酮)			71-58-9		6.17		387.2		285.4^*^		80		24	[M+H]^+^
										327.3		80		20	[M+H]^+^
40	budesonide (布地奈德)			51333-22-3		6.36		475.2		339.3^*^		-40		-31	[M-H]^-^
										357.3		-40		-20	[M-H]^-^
41	ethinylestradiol (炔雌醇)			57-63-6		6.44		295.1		145.0^*^		-40		-51	[M-H]^-^
										159.1		-40		-44	[M-H]^-^
42	diethylstilbestrol (己烯雌酚)			56-53-1		7.05		267.3		237.1^*^		-40		-42	[M-H]^-^
										251.0		-40		-36	[M-H]^-^
43	dienestrol (双烯雌酚)			84-17-3		7.06		265.2		92.7^*^		-32		-50	[M-H]^-^
										171.1		-28		-30	[M-H]^-^
44	hexestrol (己烷雌酚)			84-16-2		7.08		269.1		118.8^*^		-47		-59	[M-H]^-^
										132.8		-47		-19	[M-H]^-^
45	clobetasol propionate (丙酸倍氯他索)			25122-46-7		7.27		511.2		429.0^*^		-45		-27	[M-H]^-^
										465.1		-45		-18	[M-H]^-^

DP: declustering potential; CE: collision energy; * quantitative ion.

### 1.4 样品前处理

准确称取1.0 g燕窝样品,置于50 mL离心管中,加入8 mL水,旋涡混匀,超声处理30 min。萃取剂为15 mL乙腈-乙酸乙酯(1∶1, v/v),均质提取2 min,提取2次,室温下离心5 min,转速为4000 r/min,合并上清液,于45 ℃氮吹浓缩至近干,加入5 mL 30%甲醇水溶液溶解残渣。上样至用3 mL甲醇和3 mL水活化过的HLB小柱中,然后依次用3 mL水、3 mL 50%甲醇水溶液淋洗,弃去淋洗液,再用3 mL甲醇洗脱,收集洗脱液,并于45 ℃水浴中氮吹浓缩至近干,用1 mL 50%乙腈水溶液溶解残渣,旋涡混匀后经0.22 μm有机相滤膜过滤,滤液供LC-MS/MS测定。

## 2 结果与讨论

### 2.1 分析方法的建立

#### 2.1.1 质谱分析条件的优化

用单针进样分析目标化合物标准溶液,分别在正离子和负离子模式下进行母离子全扫描,得到各化合物的准分子离子([M+H]^+^或[M-H]^-^),比较两种模式扫描的灵敏度,选取响应值高的离子作为母离子。然后,对母离子施加一定的碰撞能量进行轰击,获得其相应的离子碎片,选择信号较强、干扰较小的两对子离子分别作为定量、定性离子。最后通过优化碰撞能量,使每种化合物的特征碎片离子对强度达到最大。优化后的质谱条件见表1。

#### 2.1.2 色谱条件的优化

在文献^[31⇓⇓⇓-35]^的基础上,实验对比了Thermo Accucore-C_18_(100 mm×2.1 mm, 2.6 μm)和Phenomenex Kinetex C_18_(100 mm×2.1 mm, 2.6 μm)色谱柱对待测物分离的效果。发现采用Phenomenex Kinetex C_18_色谱柱时,待测物色谱峰的保留时间、分离度以及对称性都更优。因此本方法选择Phenomenex Kinetex C_18_色谱柱来分离待测物。

在正离子模式下,对比了水-乙腈、0.1%甲酸水溶液-乙腈作为流动相时待测物的分离效果。结果表明,流动相甲酸化后有利于[M+H]^+^的形成,目标化合物的响应明显增强。在负离子模式下,比较了水-乙腈、0.1%氨水溶液-乙腈为流动相时的分离效果,发现流动相添加氨水后对[M-H]^-^的形成并无促进作用。因此,在正、负离子模式下,分别采用0.1%甲酸水溶液-乙腈、水-乙腈为流动相。45种化合物的总离子流色谱图(TIC)见图1。

**图1:**
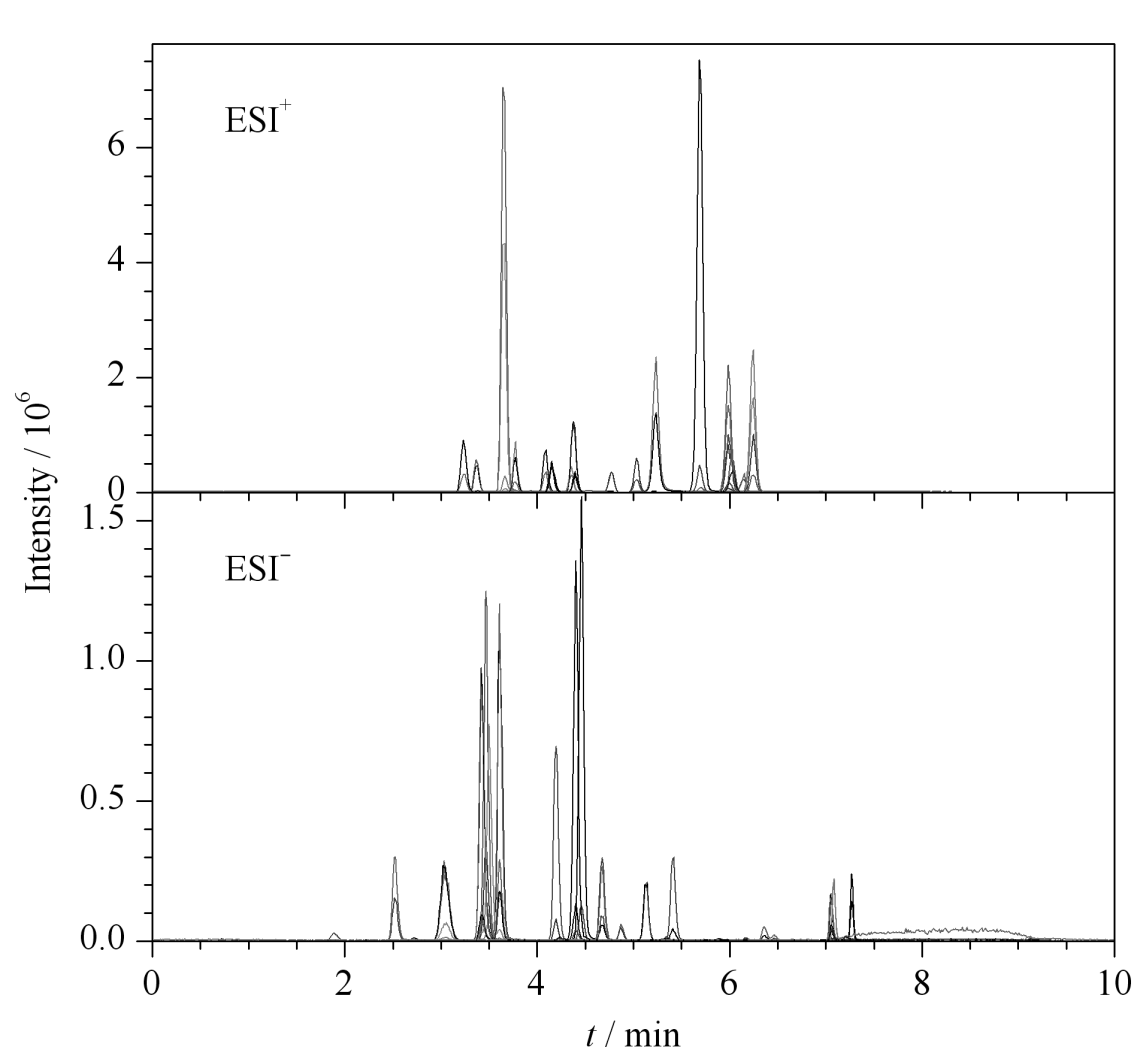
45种激素的总离子流色谱图

#### 2.1.3 提取溶剂的选择

根据现有的文献方法^[31⇓⇓⇓-35]^,结合目标化合物的性质,实验比较了乙腈、甲醇、0.1%乙酸乙腈、乙腈-乙酸乙酯(1∶1, v/v)等4种不同提取溶剂对45种激素(10 μg/kg)的提取效果。结果表明,当提取溶剂为乙腈-乙酸乙酯(1∶1, v/v)时,甲睾酮的回收率为65%、布地奈德为69%,其他43种目标物的回收率为74%~92%。而以乙腈、甲醇、0.1%乙酸乙腈为提取溶剂时,目标物回收率分别为34%~64%、26%~59%和42%~70%。故本文选择乙腈-乙酸乙酯(1∶1, v/v)作为提取溶剂。

### 2.2 基质效应

与其他分析手段不同的是,质谱分析尤其是以电喷雾电离为离子源的质谱分析可能会存在基质效应。燕窝是天然材料,基质中的杂质会对目标化合物的分析产生一定干扰,影响分析的准确度、灵敏度和精密度。本实验选取了燕窝基质溶液与50%乙腈水溶液分别配制相同浓度的标准溶液。采用公式ME=(基质匹配曲线的斜率/溶剂标准曲线的斜率-1)×100%计算基质效应^[28,33]^。基质效应为负值时,表示存在基质抑制效应;基质效应为正值时,表示存在基质增强效应。基质效应的研究结果表明,醛固酮、醋酸氟轻松、炔诺酮、地塞米松、醋酸甲羟孕酮表现为增强效应;其他40种化合物均表现为抑制效应,其中21-羟基孕酮、17*α*-羟基孕酮、康力龙等3个分析目标物的ME>15%,但<20%。总体评估,燕窝基质对多数分析目标物基质效应不明显,对回收率的影响在可接受范围内,考虑到检测的效率与成本,故本研究采用溶剂配制标准溶液,外标法定量。

### 2.3 方法学验证

#### 2.3.1 线性范围、检出限及定量限

配制45种激素的系列混合标准溶液,以各分析物的质量浓度(*x*, μg/L)为横坐标,各激素定量离子对的峰面积(*y*)为纵坐标绘制标准曲线,得到的线性回归方程和相关系数(*R*^2^)见表2。线性相关系数均≥0.9990,在线性范围内线性良好。通过向燕窝样品中添加45种激素考察方法的检出限(*S/N*=3)和定量限(*S/N*=10),分析目标物检出限和定量限分别为0.04~0.70 μg/kg和0.16~2.00 μg/kg。

**表2:** 45种激素的回归方程、相关系数、检出限、定量限及回收率(*n*=6)

No.	Compound	Regression equation	*R*^2^	LOD/ (μg/kg)	LOQ/ (μg/kg)	Recoveries/%			
						2.0 μg/kg		4.0 μg/kg	20.0 μg/kg
1	triamcinolone	*y*=7.98×10^3^*x*+3.26×10^3^	0.9996	0.30	0.97	78.0		85.7	89.2
2	aldosterone	*y*=2.41×10^3^*x*+3.95×10^3^	0.9996	0.31	1.02	58.5		61.3	61.6
3	betamethasone	*y*=7.82×10^3^*x*+3.36×10^3^	0.9994	0.13	0.53	87.1		86.6	89.3
4	fluoxymesterone	*y*=3.41×10^3^*x*+4.45×10^3^	0.9995	0.11	0.42	91.7		87.0	88.5
5	prednisolone	*y*=7.10×10^3^*x*+2.91×10^3^	0.9997	0.05	0.23	108.9		89.0	88.4
6	estriol	*y*=5.33×10^3^*x*+4.33×10^3^	0.9997	0.14	0.44	85.8		87.1	95.4
7	prednisone	*y*=4.21×10^3^*x*+3.46×10^3^	0.9998	0.04	0.18	91.0		80.4	88.1
8	hydrocortisone	*y*=5.41×10^3^*x*+2.89×10^3^	0.9996	0.05	0.18	91.2		89.6	87.8
9	boldenone	*y*=7.42×10^3^*x*+3.78×10^3^	0.9999	0.08	0.34	90.6		92.3	87.4
10	cortisone	*y*=3.87×10^3^*x*+3.39×10^3^	0.9992	0.04	0.19	77.9		77.1	86.6
11	19-nortestosterone	*y*=6.41×10^3^*x*+4.35×10^3^	0.9995	0.05	0.17	85.9		75.6	91.8
12	norethindrone	*y*=1.90×10^3^*x*+4.62×10^3^	0.9996	0.20	1.01	112.3		84.3	108.7
13	21-hydroxyprogesterone	*y*=6.12×10^3^*x*+3.40×10^3^	0.9996	0.08	0.37	78.0		79.8	77.7
14	6*α*-methylprednisolone	*y*=3.51×10^3^*x*+3.28×10^3^	0.9995	0.06	0.16	86.8		87.7	91.3
15	androstenedione	*y*=2.13×10^3^*x*+4.20×10^3^	0.9993	0.05	0.16	98.1		87.8	90.0
16	methyltestosterone	*y*=1.49×10^3^*x*+3.80×10^3^	0.9998	0.07	0.25	89.3		78.7	91.1
17	17*α*-hydroxyprogesterone	*y*=6.24×10^3^*x*+3.33×10^3^	0.9994	0.13	0.48	72.2		73.2	86.6
18	dexamethasone	*y*=4.21×10^3^*x*+3.41×10^3^	0.9998	0.06	0.21	102.3		86.0	100.3
19	flumethasone	*y*=8.21×10^3^*x*+3.19×10^3^	0.9996	0.08	0.17	90.4		86.7	90.6
20	beclomethasone	*y*=8.74×10^3^*x*+3.21×10^3^	0.9998	0.10	0.36	76.4		87.1	80.4
21	levonorgestrel	*y*=4.23×10^3^*x*+3.61×10^3^	0.9998	0.20	1.03	90.8		70.5	88.4
22	5*α*-androstan-17*β*-ol-3-one	*y*=5.42×10^3^*x*+3.44×10^3^	0.9990	0.61	2.00	99.5		86.2	101.6
23	triamcinolone acetonide	*y*=3.11×10^3^*x*+4.09×10^3^	0.9994	0.12	0.46	85.7		89.0	90.0
24	medroxyprogesterone	*y*=1.24×10^3^*x*+3.39×10^3^	0.9992	0.13	0.52	91.0		78.6	89.8
25	stanozolol	*y*=6.78×10^3^*x*+3.88×10^3^	0.9994	0.17	0.76	81.3		79.7	79.4
26	mesterolone	*y*=7.96×10^3^*x*+3.19×10^3^	0.9996	0.70	1.95	78.6		86.9	106.7
27	17*β*-estradiol	*y*=6.11×10^3^*x*+4.21×10^3^	0.9998	0.05	0.15	107.6		87.5	87.6
28	fluocinolone acetonide	*y*=4.64×10^3^*x*+3.73×10^3^	0.9996	0.10	0.48	40.2		51.1	62.7
29	estrone	*y*=2.16×10^3^*x*+3.02×10^3^	0.9998	0.14	0.46	87.9		88.3	89.0
30	fluorometholone	*y*=1.50×10^3^*x*+4.45×10^3^	0.9998	0.32	0.96	62.4		60.4	63.3
31	fludrocortisone acetate	*y*=6.24×10^3^*x*+4.26×10^3^	0.9996	0.31	1.00	107.9		86.2	106.9
32	17*α*-estradiol	*y*=5.96×10^3^*x*+3.62×10^3^	0.9991	0.13	0.49	90.2		86.3	97.5
33	trenbolone acetate	*y*=4.18×10^3^*x*+4.16×10^3^	0.9992	0.05	0.15	77.7		76.0	89.5
34	megestrol-17-acetate	*y*=5.78×10^3^*x*+4.31×10^3^	0.9996	0.05	0.21	90.2		88.9	89.0
35	progesterone	*y*=1.23×10^3^*x*+2.84×10^3^	0.9996	0.08	0.37	107.6		98.1	87.4
36	danazol	*y*=5.13×10^3^*x*+3.67×10^3^	0.9993	0.13	0.45	88.4		91.5	87.8
37	chloromadinone 17-acetate	*y*=2.41×10^3^*x*+4.06×10^3^	0.9996	0.10	0.52	85.5		78.5	98.5
38	melengestrol acetate	*y*=2.23×10^3^*x*+3.17×10^3^	0.9996	0.13	0.35	85.4		89.1	90.7
39	medroxyprogesterone-17-acetate	*y*=6.74×10^3^*x*+4.41×10^3^	0.9998	0.05	0.51	86.3		107.8	98.7
40	budesonide	*y*=3.62×10^3^*x*+3.27×10^3^	0.9978	0.14	0.48	60.1		62.5	63.6
41	ethinylestradiol	*y*=6.45×10^3^*x*+3.32×10^3^	0.9997	0.30	0.96	54.1		54.7	58.8
42	diethylstilbestrol	*y*=4.21×10^3^*x*+3.28×10^3^	0.9997	0.11	0.39	88.3		85.6	77.0
43	dienestrol	*y*=4.32×10^3^*x*+2.64×10^3^	0.9992	0.13	0.36	85.1		84.8	90.1
44	hexestrol	*y*=3.45×10^3^*x*+3.08×10^3^	0.9995	0.40	0.97	85.7		76.8	98.9
45	clobetasol propionate	*y*=4.12×10^3^*x*+3.40×10^3^	0.9995	0.17	0.42	86.5		85.8	109.8

*y*: peak area; *x*: mass concentration, μg/L.

#### 2.3.2 准确度和精密度

分别添加适量混合中间标准溶液至燕窝样品中,添加水平为2.0(灵敏度最差目标物的LOQ)、4.0和20.0 μg/kg,按照本文建立的方法进行测定,通过添加回收试验考察方法的准确度和精密度(*n*=6),结果见表2。醛固酮、醋酸氟轻松、氟米龙、布地奈德、炔雌醇的回收率为40.2%~63.6%,其他40种激素的平均加标回收率为72.2%~112.3%,相对标准偏差(RSD)为2.5%~11.6%, 40种分析目标物的准确度和精密度均符合残留分析要求。

在多残留分析时,涉及的化合物性质存在差异,取得统一良好的回收率有一定难度。本研究中醛固酮等5种目标物的回收率偏低,但考虑到其定量限分别为1.02、0.48、0.96、0.48、0.96 μg/kg,根据最低回收率折算(LOQ×40.2%),检出值分别为0.41、0.19、0.39、0.19、0.39 μg/kg,均高于5种化合物的检出限0.31、0.10、0.32、0.14、0.30 μg/kg,故本方法不会造成假阴性,可对醛固酮等5种目标物进行定性分析。如需准确定量可以考虑使用同位素内标、基质匹配、标准加入等方法。

### 2.4 燕窝中激素含量

#### 2.4.1 燕窝中激素含量情况

2017~2021年期间,采用本文建立的方法对1021个样品进行了测定,仅勃地酮、雄烯二酮、孕酮有检出(测定值≥LOD),其他激素水平均小于检出限。勃地酮、雄烯二酮与孕酮有检出的样品个数分别为807个(检出比率79%)、909个(检出比率89%)、1021个(检出率为100%),测定值范围分别为0~0.096、0~1.77、0.097~3.58 μg/kg。将检出的测定值分成5等,统计每一等所占比例,结果见图2。检出的结果中,所有样品中勃地酮测定值均<0.1 μg/kg; 764个样品中雄烯二酮测定值<1.0 μg/kg; 888个样品中孕酮测定值<1.0 μg/kg。所有样品中检出的激素含量均未超过5 μg/kg。此外,本次研究未检出国家或地区禁止的类固醇激素。

**图2:**
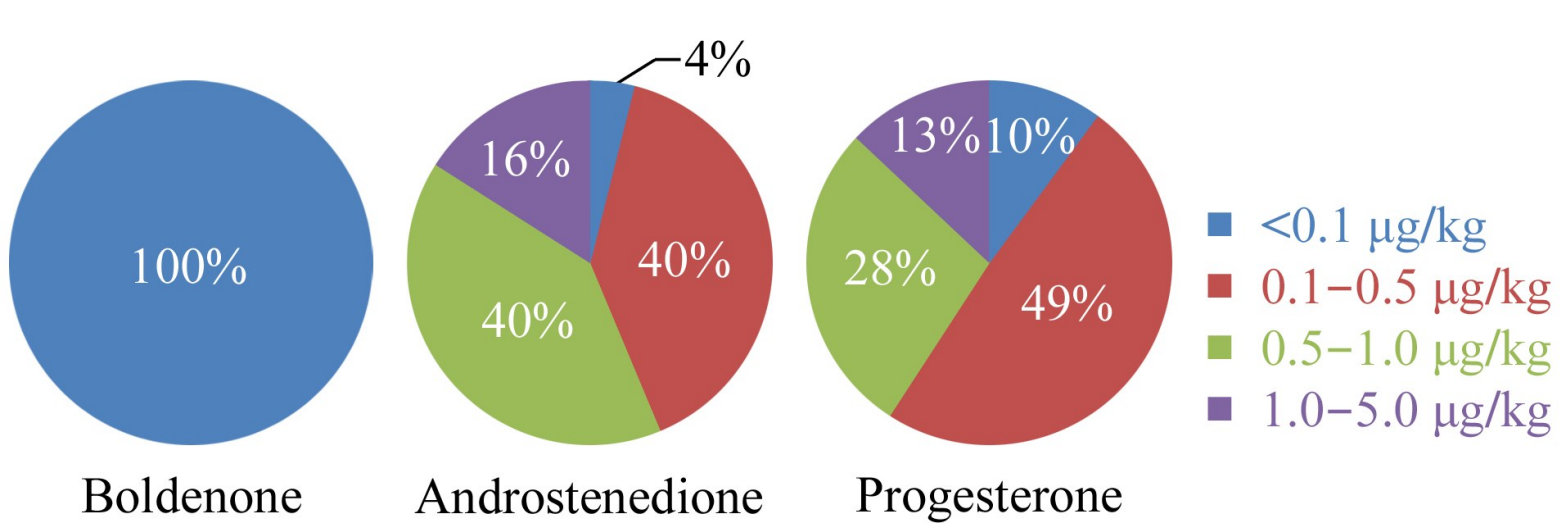
燕窝中勃地酮、雄烯二酮和孕酮的含量分布

#### 2.4.2 燕窝中检出激素的质谱解析

勃地酮、雄烯二酮与孕酮的相对分子质量分别为286.4、286.4和314.5,其形成的[M+H]^+^通过碰撞池时发生裂解生成产物离子,见图3。勃地酮的[M+H]^+^丢失一个H_2_O,得到*m/z*为269.1的二级离子,丢失质量数为152(C_10_H_16_O)的中性碎片,得到*m/z*为135.0的二级离子,丢失质量数为166(C_11_H_18_O)的中性碎片,得到*m/z*为121.0的二级离子。雄烯二酮的[M+H]^+^丢失一个H_2_O,得到*m/z*为269.2的二级离子,丢失质量数为178(C_12_H_18_O)的中性碎片,得到*m/z*为109.1的二级离子,丢失质量数为190(C_13_H_18_O)的中性碎片,得到*m/z*为97.1的二级离子。孕酮的[M+H]^+^丢失一个H_2_O,得到*m/z*为297.3的二级离子,丢失质量数为206(C_14_H_22_O)的中性碎片,得到*m/z*为109.0的二级离子,丢失质量数为218(C_15_H_22_O)的中性碎片,得到*m/z*为97.0的二级离子。

**图3:**
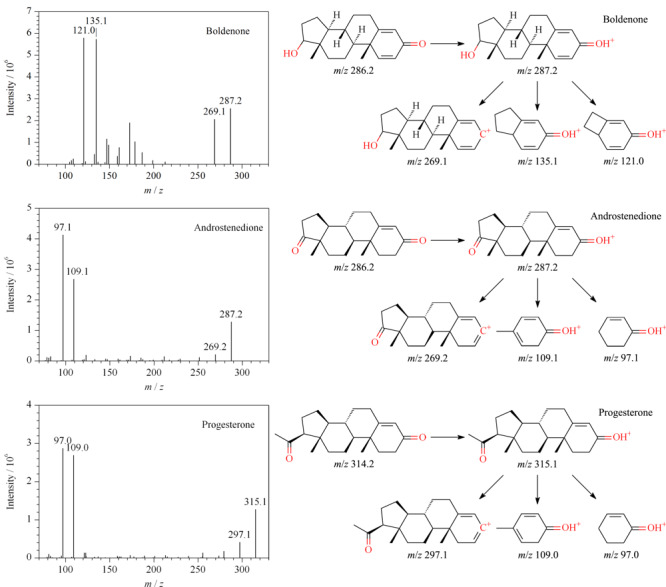
勃地酮、雄烯二酮、孕酮的二级碎片质谱图和裂解示意图

#### 2.4.3 燕窝、乳制品与鸡蛋中激素水平比较分析

随着人类活动范围越来越大,人工合成激素被大量使用并通过环境传播或食物链富集导致食品污染,即便在非直接接触的食品中亦可检出其存在,且通过食品加工措施并不能改变其含量和性质,直接或间接影响到人体生理代谢。

本研究按GB/T 21981-2008方法对市售的16批纯牛奶、70批乳制品以及15批鸡蛋中的勃地酮、雄烯二酮和孕酮进行测定,结果见表3。 勃地酮在4类产品中的含量差别不大,均为微量;15批鸡蛋中均检出雄烯二酮,含量比其他3类产品略高;孕酮含量在鸡蛋中最高,其次是纯牛奶,燕窝中含量最低,部分乳制品中未检出,可能是产品含乳量低的原因。

**表3:** 不同动物源食品中勃地酮、雄烯二酮和孕酮的含量

Product	Boldenone/ (μg/kg)	Androstenedione/ (μg/kg)		Progesterone/ (μg/kg)	
EBN	0-0.096	0	-1.77	0.097-	3.58
Pure milk	0-0.20	0	-1.88	1.82-	32.9
Dairy	0-0.14	0	-1.43	0-	20.1
Eggs	0-0.25	1.86	-5.36	16.9-	36.2

以检出的3种激素中含量最高的孕酮的平均含量进行分析,常人每人每天燕窝食用量为5 g^[36]^,鸡蛋为40~50 g^[37]^,牛奶为300 g^[37]^,根据安全风险评估方法^[38]^,估算出每人每天孕酮的摄入量为牛奶(3.54 μg/d)>鸡蛋(1.09 μg/d)>燕窝(0.0030 μg/d),由此可知食用燕窝时激素的摄入量远低于食用牛奶或鸡蛋时激素的摄入量,对健康的影响较小。

## 3 结论

本文建立了燕窝中45种激素的HPLC-MS/MS分析方法,对醛固酮、醋酸氟轻松、氟米龙、布地奈德、炔雌醇可进行定性分析,对其他40种激素可进行定性定量分析。该方法的线性范围、检出限、回收率及精密度等方法学指标均满足燕窝中40种激素的定性定量分析要求,且方法简便快速,溶剂用量少,激素检测的种类广、数量多,可望为燕窝中激素的监测和风险评估提供方法学基础。采用本研究建立的方法对燕窝中激素进行监测,结果表明燕窝中激素含量水平低于蛋与乳制品,对健康影响较小。

## References

[1] Mei X M, Wu X X, Qiao L, et al. Modern Food Science and Technology, 2020, 36(2): 277

[2] Dai Y W, Cao J, Wang Y Y, et al. Food Res Int, 2021, 140: 109875 33648193 10.1016/j.foodres.2020.109875

[3] Hou Z P, Imam M U, Ismail M, et al. Biosci Biotechnol, Biochem, 2015, 79(10): 1570 26057702 10.1080/09168451.2015.1050989

[4] Zhao R, Li G, Kong X J, et al. Drug Des, Dev Ther, 2016(10): 371 10.2147/DDDT.S88193 PMC4727516 26855562

[5] Dong J H, Tian Q J, Duan S F, et al. Food and Fermentation Industries, 2019, 45(17): 73

[6] Albishtue A, Yimer N, Zakaria M Z A, et al. Veterinary World, 2019, 12(7): 1013 31528026 10.14202/vetworld.2019.1013-1021 PMC6702562

[7] Hou Z P, Imam M U, Ismail M, et al. Food & Funct, 2015, 6(5): 1701 10.1039/c5fo00226e 25920003

[8] Haghani A, Mehrbod P, Safi N, et al. BMC Complement Altern Med, 2017, 22(17): 1498 10.1186/s12906-016-1498-x PMC5216576 28056926

[9] Haghani A, Mehrbod P, Safi N, et al. J Ethnopharmacol, 2016, 185: 327 26976767 10.1016/j.jep.2016.03.020

[10] Yew M Y, Koh R Y, Chye S M, et al. Food Biosci, 2019(32): 100466

[11] Zhang Y D, Imam M U, Ismail M, et al. J Diabetes Res, 2015, 2015: 1

[12] Ma F C, Liu D C. Asian J Chem, 2012, 24(1): 117

[13] Tong X, Hu B Y, Chen X C, et al. Journal of Zhejiang University (Agriculture & Life Science), 2017, 43(6): 734

[14] Adeel M, Song X M, Wang Y Y, et al. Environ Int, 2017, 99: 107 28040262 10.1016/j.envint.2016.12.010

[15] Wang Z W, Yan R X, Liao S W, et al. Appl Surf Sci, 2018, 457: 323

[16] Lu X, Sun J D, Sun X L, et al. TrAC-Trends Anal Chem, 2020, 127: 115882

[17] 17 96/22/EC

[18] Ministry of Agriculture. Announcement No. 250 of the Ministry of Agriculture and Rural Areas of the People's Republic of China. (2020-01-06) [2021-08-10]. http://www.moa.gov.cn/gk/tzgg_1/gg/202001/t20200106_6334375. http://www.moa.gov.cn/gk/tzgg_1/gg/202001/t20200106_6334375

[19] Matraszek-Zuchowsha I, Wozniak B, Posyniak A. Food Anal Methods, 2017, 10: 727

[20] Wolecki D, Caban M, Pazdro K, et al. Talanta, 2019, 200: 316 31036191 10.1016/j.talanta.2019.03.062

[21] Li J H, Jiang J B, Zhao D, et al. J Alloy Compound, 2018, 769: 566

[22] Özcan A, Topcuoullai D. Sensor Actuat B, 2017, 250: 85

[23] Wu H Y. [MS Dissertation]. Guangzhou: Southern Medical University, 2018

[24] Wang R J, Liu Q Z, Xu N, et al. Journal of Changchun University of Chinese Medicine, 2020, 36(3): 482

[25] Razmkhah K, Sereshti H, Soltani S, et al. Food Anal Methods, 2018(11): 3179

[26] Chen X P. [MS Dissertation]. Guangzhou: Guangzhou University, 2017

[27] He P, Aga D S. Anal Methods, 2019, 11(11): 1436

[28] Danezis G P, Anagnostopoulos C J, Liapis K, et al. Anal Chim Acta, 2016, 942: 121 27720116 10.1016/j.aca.2016.09.011

[29] Goh S X L, Duarah A, Zhang L F, et al. J Chromatogr A, 2016, 1465: 9 27562415 10.1016/j.chroma.2016.08.040

[30] Li X Q, Sun H, Zhang X R, et al. Quality and Market, 2020, 271: 40

[31] Zhou Q L, Lou T T, Wang S Y, et al. Food Research and Development, 2018, 39(9): 183

[32] Cui X L, Shao B, Zhao R, et al. Chinese Journal of Chromatography, 2006, 24(3): 213 16929834

[33] Liu H C, Li N, Lin T, et al. Chinese Journal of Chromatography, 2015, 33(11): 1163 26939362 10.3724/sp.j.1123.2015.06035

[34] Han S Y, Yu H M, Song Y L, et al. Chinese Journal of Chromatography, 2018, 36(3): 285 30136507 10.3724/SP.J.1123.2017.11043

[35] Zhu W X, Liu Y F, Yuan P, et al. Chinese Journal of Chromatography, 2010, 28(11): 1031 21381418

[36] Zhejiang Food and Drug Administration. Zhejiang Provincial Standard for Processing Traditional Chinese Medicine. Hangzhou: Zhejiang Science and Technology Press, 2006

[37] Chinese Nutrition Society. Dietary Guidelines for Chinese Residents. Beijing: People's Health Press, 2017

[38] Qian Y Z, Li Y. Risk Assessment of Quality and Safety of Agricultural Products-Principles, Methods and Applications. Beijing: China Standards Press, 2007

